# The hydrological context determines the beta-diversity of aerobic anoxygenic phototrophic bacteria in European Arctic seas but does not favor endemism

**DOI:** 10.3389/fmicb.2015.00638

**Published:** 2015-07-03

**Authors:** Anne-Catherine Lehours, Christian Jeanthon

**Affiliations:** ^1^Laboratoire Microorganismes: Génome et Environnement, Clermont Université, Université Blaise PascalClermont-Ferrand, France; ^2^Laboratoire Microorganismes: Génome et Environnement, Centre National de la Recherche Scientifique, UMR 6023Aubière, France; ^3^Oceanic Plankton Group, Station Biologique, Centre National de la Recherche Scientifique, UMR 7144Roscoff, France; ^4^Oceanic Plankton Group, Station Biologique, Sorbonne Universités, Université Pierre et Marie Curie Univ Paris 06, UMR 7144Roscoff, France

**Keywords:** photoheterotrophy, aerobic anoxygenic phototrophic bacteria, Arctic, *puf*M gene, diversity

## Abstract

Despite an increasing number of studies over the last 15 years, aerobic anoxygenic photoheterotrophic (AAP) bacteria remain a puzzling functional group in terms of physiology, metabolism, and ecology. To contribute to a better knowledge of their environmental distribution, the present study aims at analyzing their diversity and structure at the boundary between the Norwegian, Greenland, and Barents Seas. The polymorphism of a marker gene encoding a sub-unit of the photosynthetic apparatus (*puf*M gene) was analyzed and attempted to be related to environmental parameters. The Atlantic or Arctic origin of water masses had a strong impact on the AAP bacterial community structure whose populations mostly belonged to the *Alpha*- and *Gammaproteobacteria*. A majority (>60%) of *puf*M sequences were affiliated to the *Gammaproteobacteria* reasserting that this class often represents the major component of the AAP bacterial community in oceanic regions. Two alphaproteobacterial groups dominate locally suggesting that they can constitute key players in this marine system transiently. We found that temperature is a major determinant of alpha diversity of AAP bacteria in this marine biome with specific clades emerging locally according to the partitioning of water masses. Whereas we expected specific AAP bacterial populations in this peculiar and newly explored ecosystem, most *puf*M sequences were highly related to sequences retrieved elsewhere. This observation highlights that the studied area does not favor AAP bacteria endemism but also opens new questions about the truthfulness of biogeographical patterns and on the extent of AAP bacterial diversity.

## Introduction

More than 10 years ago, Kolber et al. (2000) reported that AAP bacteria, harvesting light with the pigment bacteriochlorophyll *a* (BChl *a*), are ubiquitous in the coastal and open ocean. This finding inspired an increasing number of studies on this functional group and on its expected key role in the carbon cycle. However, the metabolic potential of these microorganisms is still enigmatic and their contribution to the marine carbon cycle remains poorly understood.

AAP bacteria have a phototrophic apparatus similar to their anaerobic counterparts which realize anoxygenic photosynthesis using carbon dioxide (CO_2_) as carbon source. Although it has been suggested that AAP bacteria display a light enhanced CO_2_ incorporation (Shiba, 1984; Kolber et al., 2001), all genomes sequenced so far lack genes encoding ribulose bisphosphate carboxylase/oxygenase (RuBisCO), as well as genes for other autotrophic CO_2_ fixation pathways (Fuchs et al., 2007; Swingley et al., 2007; Newton et al., 2010; Wagner-Döbler et al., 2010). Whereas light-dependent CO_2_ fixation has been demonstrated in *Roseobacter denitrificans* through the anaplerotic pathway (Tang et al., 2009), genomic and physiological evidences showed that AAP bacteria are photoheterotrophs, using both organic substrates and light for their carbon and energy requirements (Yurkov and Csotonyi, 2009). The ability of AAP bacteria to grow in the dark also raises the question of the functional role and importance of phototrophy for these microorganisms. It has been postulated that phototrophy may confer an ecological advantage to AAP bacteria in oligotrophic environments (Kolber et al., 2000); however quantitative data do not support this hypothesis (e.g., Goericke, 2002; Schwalbach and Fuhrman, 2005; Sieracki et al., 2006). Should we be surprised by these observations? The assumption of a link between trophic conditions and ecology of AAP bacteria is simplistic and biased when considering these bacteria as a homogeneous functional group. In fact, AAP bacteria are phylogenetically diverse, and ecophysiological studies performed on a few strains revealed their great diversity in terms of physiology and metabolism (Rathgeber et al., 2004). Consequently, AAP bacterial species and/or ecotypes probably exhibit a broad range of ecological niches according to their optimal growth requirements and inhabit a variety of habitats with great environmental variability (Ritchie and Johnson, 2012).

To gain a more accurate picture of the factors that govern the distribution of AAP bacteria, the diversity of their core photosynthetic marker genes was investigated in different oceanic regions (e.g., Jiao et al., 2007; Yutin et al., 2007; Lehours et al., 2010; Ritchie and Johnson, 2012), and the observed patterns have tentatively been related to environmental variables (Jiao et al., 2007; Lehours et al., 2010). Some trends are beginning to emerge and are consistent with different ecological preferences for members of this functional group. The diversity of AAP bacteria is structured along environmental gradients and is related to multifactorial parameters (Lehours et al., 2010). For example, specific clades of AAP bacteria are associated with high (or low) abundances of *Synechococcus*, total bacteria, or chlorophyll (Ritchie and Johnson, 2012). In addition to environmental variables, the geographic distance may also play an important role in the ecological structuring of AAP bacteria (Ritchie and Johnson, 2012).

In this study, we analyzed the diversity of AAP bacteria, based on the polymorphism of the M subunit of anoxygenic photosynthetic reaction center genes, along two transects through a complex region around the polar front located between the Norwegian, Barents and Greenland Seas. The polar front divides relatively warm and saline waters from Atlantic origin from colder and fresher Arctic waters. We used this peculiar environmental context and the general environmental properties that shape them to (i) identify AAP bacterial populations in this newly explored area and (ii) determine the influence of the origin of water masses and of the local environment on the AAP bacterial community structure.

## Materials and methods

### Sampling

**Figure 1:**
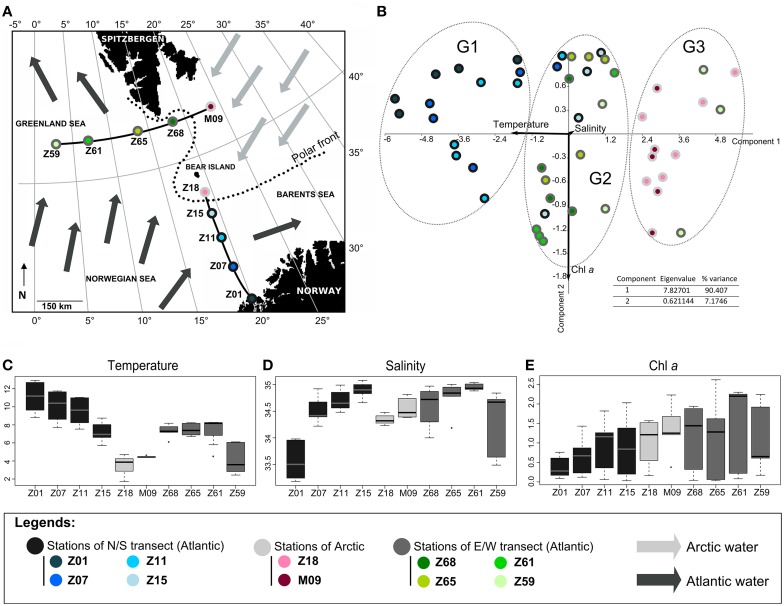
**Oceanographic context. (A)** Map of the cruise area. The two transects S/N (south/north) and E/W (east/west) are indicated. Arrows represent surface circulation and origin of water masses (adapted from Not et al., 2005); **(B)** Principal component analysis of environmental parameters. The percentage of variability and the eigenvalue are indicated. Samples (circles) and variables (arrows) are plotted against the first two axes. Variables are salinity (psu), temperature (°C) and Chl *a* (μg L^−1^). The groups identified (G1, G2, and G3) were confirmed by ANOSIM statistics (data not shown). **(C–E)** Boxplots displaying **(C)** temperature, **(D)** salinity and Chl *a* concentration.

Sampling was conducted in an area between the Norwegian, Greenland, and Barents Seas during an oceanographic cruise on board of the F/F *Johan Hjort* (Norwegian Institute of Marine Research) (Not et al., 2005). Ten stations were sampled from 20 August to 8 September 2002 along two transects (south/north, S/N and east/west, E/W) south of the Svalbard archipelago (Figure 1A). Samples were collected at several depths with a conductivity–temperature–depth (CTD) rosette system equipped with Niskin bottles (5 L). Nutrients were analyzed at the Norwegian Institute of Marine Research using standard techniques, and chlorophyll *a* (Chl *a*) concentrations were determined on board. An overview of position, bottom depth and hydrology for each sampling station was previously reported (Not et al., 2005). At all stations, water samples for DNA analysis were collected as described by Lovejoy et al. (2006). Briefly, water was prefiltered to remove large organisms and particles and microbial biomass was collected in 0.22 μm Sterivex filter units with a peristaltic pumping system. Filters were frozen at −80°C in lysis buffer (40 mM EDTA, 50 mM Tris-HCl, 0.75 M sucrose) until nucleic acids were extracted.

### DNA extraction

DNA extraction was performed as described by Lovejoy et al. (2006). Briefly, following enzymatic digestion [lysozyme (1 mg mL^−1^) and proteinase K (0.21 mg mL^−1^)], lysates were recovered and nucleic acids were extracted twice [phenol-chloroform-isoamyl alcohol (25:24:1), followed by chloroform-isoamyl alcohol (24:1)], and were concentrated using Centricon-100 concentrators (Millipore).

### Diversity of the *puf*M gene

Ten clone libraries were constructed using pufM_forward (5′-TACGGSAACCTGTWCTAC-3′, Béjà et al., 2002) and pufM_WAW primers (5′-AYNGCRAACCACCANGCCCA-3′, Yutin et al., 2005) to amplify partial sequences of the *puf*M gene (245-bp fragments). The following PCR conditions were used: 95°C for 5 min followed by 40 cycles of 95°C for 30 s, 58°C for 30 s, and 72°C for 30 s, using a final extension step at 72°C for 10 min. PCR reactions (50 μL) contained 5 X buffer (10 μL), 2 mM MgCl_2_, 10 pmol of each dNTP (Eurogenetec), 10 pmol of each primer, 2.5 U of GoTaq Flexi DNA polymerase (Promega) and 50–100 ng of template DNA. Amplified products were checked by electrophoresis in 1.5% agarose in 0.5 X Tris-acetate-EDTA buffer and further quantified with a DNA quantitation fluorescence assay kit (Sigma-Aldrich). For each library, amplicons were sequenced on an ABI 3130 POP7 sequencer (Applied Biosystems) at the Biogenouest Sequencing-Genotyping Platform (Roscoff site) after being ligated to the TOPO-TA vector (Invitrogen) and cloned into *Escherichia coli* following the manufacturer's recommended protocol (Invitrogen Corporation, Carlsbad, CA). Sequences were manually curated, subjected to BLAST search against publicly available sequences, grouped into Operational Taxonomic Units (OTUs) according to a cut-off value of 94% using BOSQUE (available at http://bosque.udec.cl, Ramírez-Flandes and Ulloa, 2008). Sequence data were analyzed with the ARB software package (Ludwig et al., 2004) and phylogenetic reconstruction was performed as described previously (Lehours et al., 2010). Phylogenetic tree display and annotation were performed with iTOL software (Letunic and Bork, 2006).

Representative sequences (one for each out) are deposited in the GenBank database under accession numbers KM654564-KM654598.

### *puf*M gene profiling

Profiling of *puf*M genes was performed by Temporal Temperature gel Gradient Electrophoresis (TTGE) after PCR amplification using a Cy5-labeled pufM_forward primer and a primer pufM_WAW with a 40-bp GC-clamp (CGCCCGCCGCGCCCCGCGCCCGTCCCGCCGCCCCCGCCCG) added at the 5′-end. The electrophoresis conditions were similar as described by Lehours et al. (2010). Gel images were obtained at a 100 μm resolution using a Typhoon Trio variable mode imager (Amersham Biosciences, Piscataway, NJ). Typhoon scans were acquired using the 633 nm excitation laser and the 670 BP 30 emission filter as recommended by the manufacturer for the detection of Cy5-labeled molecules. All gels were scanned with photomultiplier tube voltages to maximize the signal without saturating fingerprint bands. Band patterns were analyzed using GELCOMPARE 6.5 software package (Applied Maths, Kortrijk, Belgium). In band assignment, a 1% band position tolerance (relative to the total length of the gel) was applied, which indicates the maximal shift allowed for two bands in different TTGE patterns to be considered as identical. Richness (number of TTGE bands in a profile) and diversity (Shannon-Wiener index) were computed as previously described (Smith and Wilson, 1996).

### Statistical analyses

Box plots were performed using R software (version 2.15.2) available at http://cran.univ-lyon1.fr/bin/windows/base. Principal Component Analyses (PCA), Canonical Correspondence Analyses (CCA) and correlations were performed using PAST 1.81 (Hammer et al., 2001) available at http://palaeo-electronica.org/2001_1/past/issue1_01.htm.

## Results and discussion

### Brief overview of the hydrological context

The PCA, including temperature, salinity and Chl *a* as variables, explained a huge part of the variance between stations. It mainly discriminated waters of the N/S transect from those of the E/W transect and grouped separately Arctic waters (Figure 1B). The PCA well illustrated the temperature gradient existing between stations (Figures 1B,C). Since salinity and chlorophyll a (Chl *a*) were locally highly variable with depth, they did not discriminate waters of Arctic or Atlantic origin (Figures 1B,D,E) as summarized below (for detailed information on the hydrological context, see Not et al., 2005).

Waters of Arctic origin (stations Z18 and M09) were characterized by low temperatures (range 1.78°C–4.78°C), low salinities (ca. 34.5 psu) (Figures 1C,D) and showed high surface Chl *a* concentrations (>1.4 μg L^−1^ and >0.8 μg L^−1^, respectively) (Figure 1E). The Atlantic-influenced waters [stations Z01 to Z15 (S/N transect) and stations Z59 to Z68 (E/W transect)] were characterized by higher surface temperatures (> 6°C) and salinities generally above 34.5 psu (Figures 1B,C). Chl *a* concentrations were lower (<1.2 μg L^−1^ and <0.5 μg L^−1^, respectively) (Figure 1E). Differences between stations influenced by Atlantic waters were identified. The station Z01 with low surface salinity had coastal features whereas low temperature and salinity at station Z59 probably reflected the influence of Arctic waters flowing south along the east coast of Greenland and directed eastward by one of the Greenland Sea gyres (see details in Not et al., 2005). Accordingly, the station Z59 grouped with Arctic stations Z18 and M09 (Figure 1B). Stations Z65 and Z68, located on the E/W transect at the intersection of the Arctic and Atlantic waters (polar front), exhibited intermediate temperatures, salinities and Chl *a* concentrations. Station Z15, close to the polar front, was slightly colder and saltier than other stations of the N/S transect explaining its clustering with stations of the E/W transect (Figure 1B).

### The partitioning of water masses drives the beta diversity of AAP bacteria

Considering the hydrological context of the sampled region, 3 hypotheses could be drawn about the distribution of AAP bacterial populations: (H1) different patterns in relation to the S/N, E/W transects and Arctic waters might be observed in AAP bacterial community composition, (H2) the sampled area being a frontier zone where Atlantic and Arctic waters meet, its AAP bacterial community composition should reflect that of the water masses, and (H3) the distribution of AAP bacterial populations should be dependent on the environmental local context (e.g., nutrient concentrations) which is highly variable with depth (Table S1, Not et al., 2005).

**Figure 2:**
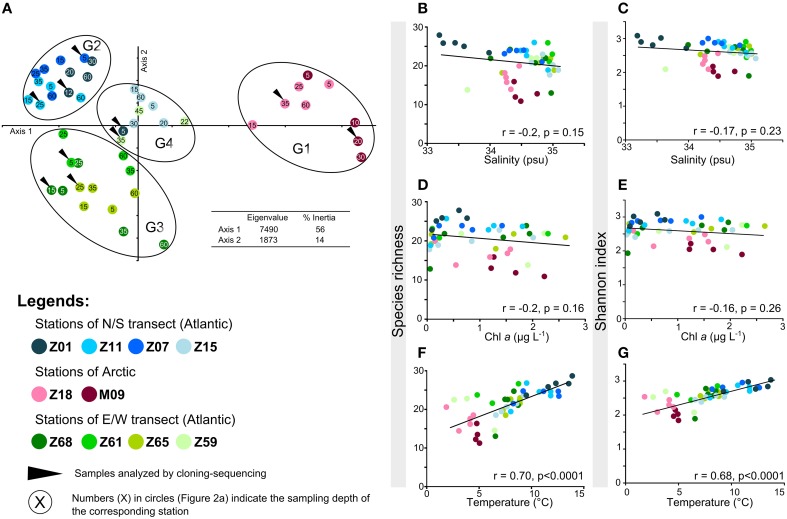
**Alpha- and beta-diversity of AAP bacteria according to TTGE band patterns. (A)** Principal component analysis performed using the relative intensity peaks of the TTGE bands. The groups identified (G1, G2, G3, and G4) were confirmed by ANOSIM statistics (data not shown). **(B–F)** Plots of the correlation between AAP richness and **(B)** salinity (psu), **(D)** Chl*a* (μ g L^−1^), **(F)** temperature (°C) and between AAP diversity (expressed using the Shannon-Wiener index) and **(C)** salinity, **(E)** Chl*a*, **(G)** temperature.

To determine the distribution of AAP bacterial populations, the polymorphism of a 245-bp region targeted on the *puf*M gene was analyzed by TTGE profiling. Among the 4 groups identified by PCA (Figure 2A), G1 included samples of Arctic-influenced waters, and G2 and G3 contained stations of the S/N and E/W transects, respectively. This may indicate very different environmental conditions, although such differences were not clearly reflected by the individual ancillary parameters (Table S1). Moreover, these parameters were locally highly variable with depth (see stations Z15 and M09 as examples, Table S1) without affecting the distribution of AAP bacterial populations.

The temperature gradient observed between stations (Figure 1B) could drive to a distribution of AAP bacterial populations according to their physiological preferences. But as this parameter also reflects the origin of oceanic water masses (Figure 1B), the hypothesis H1 is not null. The assumption that the origin of water masses strongly impacts the distribution of AAP bacterial populations is reinforced by the discrimination of a fourth group on the PCA. The group G4 mainly includes samples from the stations Z15 and Z59 (Figure 2A). These stations that are close to the polar front (Z15) and influenced by Arctic waters (Z59) may contain AAP bacterial communities formed by populations originating from both Arctic and Atlantic water masses. Without ruling out the hypothesis H3, our analysis demonstrates that the origin of water masses has a strong impact in structuring AAP bacterial communities. Whereas a similar observation was reported by Boeuf et al. (2013) in the Beaufort Sea, this was not the case in other environments. For example, stratification of water masses was shown to be a critical factor determining the distribution of AAP bacterial populations in the Mediterranean Sea, with synergetic driving forces (e.g., nutrients, light) explaining a great part of the variability of AAP bacterial assemblages (Lehours et al., 2010). However, we cannot exclude that some specific AAP bacterial populations can be associated with specific environmental parameters as observed in the Pacific Ocean (Ritchie and Johnson, 2012).

### Temperature is a good predictor of the alpha diversity of AAP bacteria

In the sampled region, trophic conditions, considering Chl *a* as an index of the productivity, and salinity gradients were not major determinants affecting the alpha diversity of AAP bacteria (Figures 2B–E). These observations contrast with previous studies where waters with higher productivity displayed lower AAP bacterial diversity than oligotrophic waters (Jiao et al., 2007). AAP bacteria were also reported to be influenced by changes in salinity (e.g., Waidner and Kirchman, 2008; Boeuf et al., 2013), but such relationship was not found in the present study. Although salinity varied along transects (from 33.2 to 35.1 psu) (Table S1, Figure 1D), these variations probably remained in a range of physiological tolerance for the corresponding AAP populations.

The data presented here demonstrate strong correlations between temperature and AAP bacterial diversity and richness (Figures 2F,G). The relationship is linear, with highest richness and Shannon diversity at highest temperatures (Figures 2F,G). The data are consistent with theories predicting that low diversity at extreme temperatures is directly related to temperature stress, and that fewer species are adapted to this stress. Indeed, temperature has a strong influence on whether a given organism can survive and/or thrive, which is both indirect, through its influence on water, and direct, through its influence on the organic molecules composing the living cells (Poindexter, 2009). We conclude that temperature is a good statistical predictor of AAP diversity, although this may be due to co-variance with physical and/or chemical factors that were not measured in the present study.

### Alpha- and gammaproteobacteria dominate the AAP community

**Figure 3:**
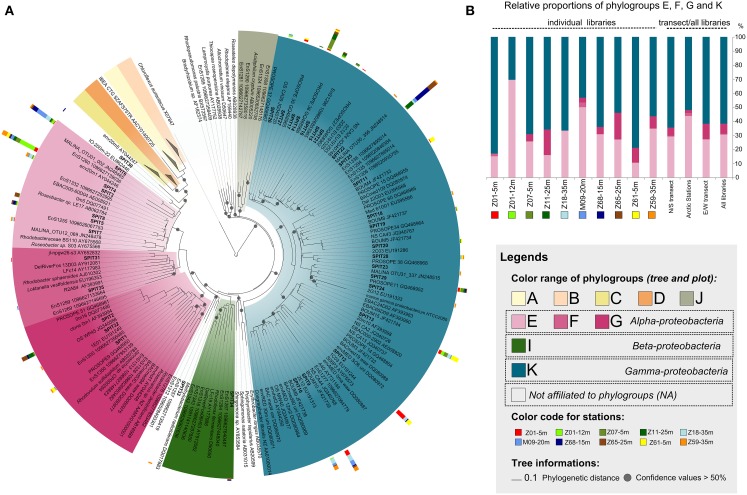
**Diversity and distribution of AAP bacteria along the sampled transects. (A)** *puf*M phylogenetic tree showing the inferred phylogenetic relationships of *puf*M gene sequences. Color ranges highlight the different phylogroups defined by Yutin et al. (2007). Tree is based on a neighbor-joining (NJ) tree to which short sequences were added by ARB_PARISMONY. Circles on nodes represent confidence values >50% for branches found in the initial NJ tree. Sequences retrieved in this study are indicated as SPIT1 to SPIT35. The multivalue bar charts represent the relative frequencies of the corresponding OTU in the clone libraries. Samples are labeled as follows: Station-Depth. Colors used to represent the clone libraries are indicated at the left of the tree. **(B)** Relative proportions of the phylogroups defined by Yutin et al. (2007) in individual libraries, in both transects, and in all stations.

To further examine the complexity of the AAP bacterial community, 10 of the 44 samples, including 2–3 representatives of each group identified by the PCA (Figure 2A), were selected for phylogenetic analyses. With the exception of the Z59-35 m library, coverage values (>79%) indicated that most of the diversity was detected in the libraries (Table S1). All the 341 sequences were aligned, subjected to phylogenetic analyses and 35 distinct OTUs were identified after grouping the sequences at 94% nucleic acid sequence similarity (Table S2). Similarity-based OTUs did not contain sequences with mixed phylogenetic signal and all were monophyletic (data not shown). Similarly to previous observations in other oceanic regions (e.g., Yutin et al., 2007; Lehours et al., 2010; Jeanthon et al., 2011; Ritchie and Johnson, 2012; Boeuf et al., 2013), most AAP bacteria belonged to the *Alpha*- and *Gammaproteobacteria* classes (Figures 3A,B, Table S2).

More than 60% of the *puf*M sequences were affiliated to phylogroup K within the *Gammaproteobacteria* (Figure 3A) reasserting that members of this group represent major components of the AAP bacterial community in diverse oceanic regions. Indeed, they dominate in the Atlantic Ocean, the Baltic Sea, and the North Pacific Ocean (Hu et al., 2006; Mašín et al., 2006; Yutin et al., 2007; Boeuf et al., 2013) with a record in their relative contribution to AAP bacteria assemblages in the Mediterranean Sea in summer (Lehours et al., 2010; Jeanthon et al., 2011). The absence of phylogroup K in high-latitude (>60°N) polar waters (Boeuf et al., 2013 and >60°S, Koh et al., 2011) is an exception to this global trend, suggesting that its members are unable to thrive in ice-cold marine waters. In this study, their prevalence in Arctic-influenced waters (stations Z18, M09, and Z59) demonstrate that they are however well adapted to slightly positive temperatures.

The overall relative proportion of the alphaproteobacterial groups E, F, and G closely related to the *Rhodobacteraceae* was >30% (Figures 3A,B, Table S2). Among them, phylogroup E was the most widely represented as previously reported in other marine systems (e.g., Yutin et al., 2007; Salka et al., 2008; Boeuf et al., 2013). Whereas alphaproteobacterial groups E and G are known to favor nutrient-rich conditions (Lehours et al., 2010; Ferrera et al., 2014), association with phytoplankton blooms (González et al., 2000) and coastal regions (Lehours et al., 2010; Ritchie and Johnson, 2012), their relative proportions were not related to the trophic state of the environment (Figures 3A,B, Tables S1, S2). This indicates that factors other than those generally thought may prevail in the regulation of their abundance. Interestingly, SPIT6 the second most abundant alphaproteobacterial OTU was identical (98% identity) to an OTU abundantly found in the Beaufort, Chukchi and Bering Seas and still present in the North Pacific Ocean (Boeuf et al., 2013). Its large distribution along the two transects with highest contributions near the polar front is in accordance with these earlier findings, supporting a preference for high-latitude biomes.

About 17% of the *puf*M sequences were highly similar (≥98%) to that of *Roseobacter* sp. LE17 (Figure 3A, Table S2). The *Roseobacter* lineage represents up to 25% of marine bacterial communities, especially in coastal and polar regions (Acinas et al., 2004). Similar *Roseobacter* contributions were observed in other oceanic regions (Ritchie and Johnson, 2012; Boeuf et al., 2013). Cultivated bacterial species are rarely detected in molecular diversity surveys based on 16S rRNA genes (Pace, 1997) and cultivation of the most abundant bacteria in the environment remains a challenging prospect for microbial ecologists. Therefore, the high proportion of sequences related to cultivated alphaproteobacterial species in different biomes supports the idea that these AAP bacteria are probably less affected by the cultivation bias than other bacterial groups. This may be related to the copiotrophic mode of life and the nutritional flexibility (Newton et al., 2010) they display in the environment by quickly responding to dissolved organic carbon released from phytoplankton blooms. *Gammaproteobacteria* from phylogroup K share the same habitats than their more readily cultivable alphaproteobacterial counterparts. However, only few strains have been obtained using the culture conditions and carbon-rich media that allowed the isolation of alphaproteobacterial AAPs. This suggests that they have physiological traits distinct from copiotrophs and are probably better adapted to low and high nutrient availability of the marine environment.

### The hydrological context determines the beta diversity of AAP bacterial populations

The relative proportions of *Alpha*- and *Gammaproteobacteria* were not related to the partitioning of water masses (Figure 3B). This is not surprising because AAP bacteria are not a homogeneous functional group, either at the overall or at of the phylogroup level. The ecological niches of AAP bacteria may be delimited at the species and/or ecotype levels, although the term “species” is probably outlier for them.

**Figure 4:**
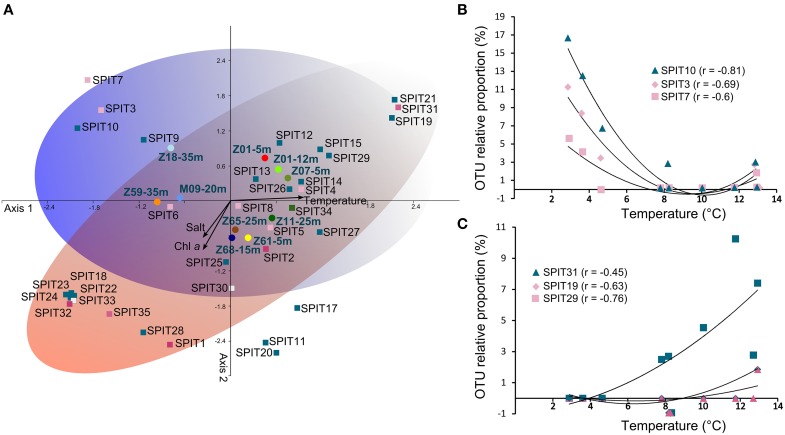
**Relationship between relative abundance of OTUs and environmental parameters. (A)** Canonical correspondence analysis performed using relative abundance of OTUs. OTUs (squares), stations (circles), and variables (arrows) are plotted against the first two axes. Samples are labeled as follows: Station-depth. Colors used to represent stations are identical to those in the Figure 3. OTUs are colored according to their phylogroup affiliation (see Figure 3). The blue and red ellipses illustrated the temperature and salinity/trophic gradient, respectively. Plots of the correlation between the relative abundance of SPIT19, SPIT29, and SPIT31 **(B)** and of SPIT3, SPIT7, and SPIT10 **(C)** with temperature. All the correlations are significant (*p* < 0.05).

A canonical correspondence analysis (CCA) was performed using relative proportions of OTUs to determine if specific AAP bacterial populations are associated with specific environmental parameters. Consistent with the clustering deduced from TTGE analyses the sampled stations grouped in the CCA according to Atlantic or Arctic water masses (Figure 4A) reinforcing the notion that the structure of AAP populations at a fine phylogenetic scale is closely related to the partitioning of water masses. This beta-diversity pattern of AAP bacteria is supported by observation of AAP populations exhibiting a clear preference for some hydrological features, notably temperature preferences (Figures 4A–C). For example, SPIT19, SPIT29, and SPIT31 seem to thrive in warmer waters (Figure 4B). Positive or negative significant correlations (*p* < 0.05) were also found between Chl*a* concentrations and the relative abundance of SPIT1 (*r* = 0.62), SPIT28 (*r* = 0.71), SPIT29 (*r* = −0.6), but also between salinity and the relative abundance of SPIT19 (*r* = −0.91), SPIT21 (*r = −0.65)* and SPIT31 (*r = −0.65)*. However, understanding the factors that govern the environmental distribution AAP bacteria is not so simple, as illustrated by the relationship between the relative abundances of OTUs SPIT3, SPIT7, and SPIT10 and temperature (Figure 4C). The significant (*p* < 0.05) negative correlation observed suggests that the corresponding AAP populations are favored by colder waters (Figure 4B). However, their increased occurrence at temperatures >12°C (Figure 4C) and their phylogenetic affiliation with *puf*M sequences retrieved from warmer biomes (Figure 3A, Table S2) illustrated that AAP populations probably exhibit an ecotypic differentiation as postulated previously (Waidner and Kirchman, 2008; Lehours et al., 2010).

### *puf*M sequences retrieved in the present biome are highly similar to sequences from other oceanic regions

Isolated and extreme environments, such as the present biome, are expected to be sources of novel phylotypes (Lovejoy et al., 2006). Considering that >90% of the *puf*M sequences were similar to sequences from other oceanic regions, we must acknowledge that AAP bacteria in the Norwegian and Barents Seas are not endemic. In a recent exploration of the AAP diversity in the western Arctic Ocean, *puf*M sequences of the dominant OTUs were also similar to sequences already detected elsewhere (Boeuf et al., 2013).

We acknowledge that the Arctic Ocean, as an enclosed ocean connected to the Pacific and Atlantic oceans (Jones, 2001), is not completely isolated. Therefore, pelagic bacteria may be readily disseminated by oceanic currents. Somewhat surprisingly, however, 40% of *puf*M sequences belonged to OTUs (>94% identity) retrieved from the Mediterranean Sea (Figure 3A, Table S2) whose hydrological conditions (e.g., temperature, salinity, trophic regime) clearly differ from that of the studied biome. This result contradicts the assumption that AAP bacteria in the Mediterranean Sea are different from those in open ocean waters (Lehours et al., 2010) and raises also the question on the congruence in biogeographical patterns of AAP bacteria following on the Baas-Becking hypothesis “Everything is everywhere, but, the environment selects” (Baas Becking, 1934).

### Have microbial ecologists caught almost all the diversity of marine AAP bacteria?

Investigations on the diversity of AAP bacteria are relatively recent, and although the number of sequences in public databases has greatly increased over the past 10 years, one might expect to find populations exhibiting new *puf*M OTUs in newly explored ecosystems. It could be considered that a hidden part of the AAP diversity is not accessible through classical cloning-sequencing approaches and that high-throughput sequencing methods could reveal a “rare biosphere of AAP bacteria” as most of the prokaryotic diversity is in the less abundant taxa (Sogin et al., 2006). The recent work of Ferrera et al. (2014) addressed this issue by generating about hundreds of thousands reads through pyrosequencing of *puf*M genes from seasonal water samples at a coastal oligotrophic environment of the northwestern Mediterranean Sea. Whereas pyrosequencing approaches are expected to increase the number of OTUs by at least one order of magnitude (Crespo et al., 2013), those authors identified only twice as much OTUs per sample than Lehours et al. (2010) who obtained 7–18 OTUs per sample in ~400 clones obtained along two large transects in the Mediterranean Sea.

Overall, results gathered from recent sampling expeditions and sequencing efforts suggest that a large part of the AAP bacterial diversity has already been sampled in marine environments.

## Conclusion

The present analysis of the AAP bacterial diversity demonstrated conclusively that variation of AAP bacterial populations across transects was much greater than along depth profiles, contrasting with previous observations in other oceanic provinces. Temperature was found to have a major role in structuring AAP bacterial populations in the present biome but as this factor is linked to water mass origin, other physical and/or chemical variables may also be contributing and synergetic factors. Understanding the factors that govern AAP bacteria diversity patterns is therefore not simple as these factors seem dependent of the oceanographic context. Surprisingly, most *puf*M sequences were similar to sequences retrieved elsewhere with, for example, a large number of gammaproteobacterial AAP sequences closely related to sequences from the Mediterranean Sea. These observations highlighted several issues to be addressed, such as the existence of biogeographical patterns for AAP bacteria and the yet known extent of their diversity in marine systems. These questions, which cannot be resolved on the basis of this study, will need to rely on comprehensive analyses integrating comparable datasets of *puf*M sequences from different oceanic regions.

### Conflict of interest statement

The authors declare that the research was conducted in the absence of any commercial or financial relationships that could be construed as a potential conflict of interest.
