# Supplementary material for: The hydrological context determines the beta-diversity of aerobic anoxygenic phototrophic bacteria in European Arctic seas but does not favor endemism
Source: Front Microbiol. 2015 Jul 3;6:638. doi: 10.3389/fmicb.2015.00638 (PMC4490794; doi:10.3389/fmicb.2015.00638)
Supplement: Supplementary file 1 [file Table1.DOCX]

| Table S1. Main characteristics of the stations sampled. Richness and diversity of AAP bacteria according to TTGE band patterns are indicated. The number of OTUs and the coverage value for each clone library are also indicated. Nd, Not determined. | | | | | | | | | | | | | | | | |
| --- | --- | --- | --- | --- | --- | --- | --- | --- | --- | --- | --- | --- | --- | --- | --- | --- |
|  |  |  |  |  |  |  |  |  |  |  |  |  |  |  |  |  |
|  | | | | | | | | | | | | | | | | |
|  | | | | | | | | | | | | | | | | |
|  |  |  |  |  |  | **Ancillary parameters** | | | | | | | **TTGE band patterns** | | **Cloning-sequencing analyses** | |
|  | **Station** | **Latitude** | **Longitude** | **DCM (m)** | **Depth (m)** | **Temp. (°C)** | **Salinity (psu)** | **Chla (µM)** | **Nitrite (µM)** | **Nitrate (µM)** | **Phosphate (µM)** | **Silicate (µM)** | **AAP richness** | **AAP diversity** | **No. of OTUs** | **Coverage** |
| **Stations of N/S transect (Atlantic)** | **Z01** | 70.5007 | 20.0218 | 13 | 5 | 12.92 | 33.18 | 0.61 | 0.02 | 0.04 | 0.06 | 0.39 | 28 | 3.1 | 14 | 87 |
|  |  | 70.5007 | 20.0218 | 13 | 12 | 12.70 | 33.25 | 0.76 | 0.04 | 0.15 | 0.09 | 0.52 | 26 | 2.9 | 11 | 81 |
|  |  | 70.5007 | 20.0218 | 13 | 20 | 11.51 | 33.44 | 0.28 | 0.16 | 1.1 | 0.18 | 1.09 | 26 | 2.8 | Nd | Nd |
|  |  | 70.5007 | 20.0218 | 13 | 30 | 10.87 | 33.57 | 0.17 | 0.3 | 2.43 | 0.28 | 1.57 | 25 | 3 | Nd | Nd |
|  |  | 70.5007 | 20.0218 | 13 | 60 | 8.81 | 33.98 | 0.09 | Nd | Nd | Nd | Nd | 23 | 2.7 | Nd | Nd |
|  | **Z07** | 71.501 | 19.8025 | 25 | 5 | 11.74 | 34.42 | 0.67 | 0.02 | 0.02 | 0.05 | 0.07 | 24 | 2.9 | 12 | 87 |
|  |  | 71.501 | 19.8025 | 25 | 15 | 11.75 | 34.22 | 0.87 | Nd | Nd | Nd | Nd | 23 | 2.9 | Nd | Nd |
|  |  | 71.501 | 19.8025 | 25 | 25 | 10.40 | 34.31 | 1.43 | 0.01 | 0.01 | 0.08 | 0.03 | 24 | 3 | Nd | Nd |
|  |  | 71.501 | 19.8025 | 25 | 35 | 8.62 | 34.53 | 0.23 | Nd | Nd | Nd | Nd | 24 | 2.9 | Nd | Nd |
|  |  | 71.501 | 19.8025 | 25 | 60 | 7.70 | 34.82 | 0.12 | Nd | Nd | Nd | Nd | 21 | 2.8 | Nd | Nd |
|  | **Z11** | 72.502 | 19.5728 | 25 | 5 | 11.04 | 34.56 | 1.26 | 0.01 | 0.04 | 0.08 | 0.09 | 24 | 2.8 | Nd | Nd |
|  |  | 72.502 | 19.5728 | 25 | 20 | 10.96 | 34.60 | 1.16 | 0 | 0.02 | 0.05 | 0.08 | 23 | 2.9 | Nd | Nd |
|  |  | 72.502 | 19.5728 | 25 | 25 | 10.03 | 34.48 | 1.82 | Nd | Nd | Nd | Nd | 24 | 2.9 | 11 | 91 |
|  |  | 72.502 | 19.5728 | 25 | 35 | 8.90 | 34.70 | 0.36 | Nd | Nd | Nd | Nd | 26 | 2.7 | Nd | Nd |
|  |  | 72.502 | 19.5728 | 25 | 60 | 7.53 | 34.92 | 0.06 | Nd | Nd | Nd | Nd | 19 | 2.6 | Nd | Nd |
|  | **Z15** | 73.29 | 19.18 | 16 | 5 | 8.75 | 34.83 | 1.38 | 0.02 | 0.04 | 0.11 | 0.73 | 23 | 2.6 | Nd | Nd |
|  |  | 73.29 | 19.18 | 16 | 15 | 8.03 | 34.66 | 2.03 | Nd | Nd | Nd | Nd | 21 | 2.6 | Nd | Nd |
|  |  | 73.29 | 19.18 | 16 | 20 | 7.11 | 35.00 | 0.84 | 0.27 | 6.23 | 0.5 | 2.75 | 20 | 2.6 | Nd | Nd |
|  |  | 73.29 | 19.18 | 16 | 30 | 6.69 | 35.08 | 0.2 | 0.08 | 10.98 | 0.73 | 4.15 | 19 | 2.4 | Nd | Nd |
|  |  | 73.29 | 19.18 | 16 | 60 | 6.14 | 34.94 | 0.03 | Nd | Nd | Nd | Nd | 18 | 2.5 | Nd | Nd |
| **Stations of Arctic** | **Z18** | 73.9992 | 19.2097 | 6 | 5 | 4.70 | 34.28 | 1.57 | 0.03 | 0.27 | 0.22 | 0.94 | 18 | 2.3 | Nd | Nd |
|  |  | 73.9992 | 19.2097 | 6 | 15 | 4.25 | 34.23 | 1.52 | Nd | Nd | Nd | Nd | 17 | 2.5 | Nd | Nd |
|  |  | 73.9992 | 19.2097 | 6 | 25 | 3.87 | 34.25 | 1.21 | Nd | Nd | Nd | Nd | 16 | 2.3 | Nd | Nd |
|  |  | 73.9992 | 19.2097 | 6 | 35 | 2.87 | 34.32 | 0.55 | Nd | Nd | Nd | Nd | 14 | 2.1 | 8 | 89 |
|  |  | 73.9992 | 19.2097 | 6 | 60 | 1.74 | 34.46 | 0.16 | Nd | Nd | Nd | Nd | 20 | 2.6 | Nd | Nd |
|  | **M09** | 76.3095 | 23.783 | 20 | 5 | 4.44 | 34.38 | 1.25 | 0.06 | 0.66 | 0.2 | 1.29 | 16 | 2.2 | Nd | Nd |
|  |  | 76.3095 | 23.783 | 20 | 10 | 4.41 | 34.40 | 1.68 | 0.06 | 0.67 | 0.2 | 1.28 | 12 | 2 | Nd | Nd |
|  |  | 76.3095 | 23.783 | 20 | 20 | 4.63 | 34.48 | 2.23 | 0.07 | 0.98 | 0.2 | 1.28 | 11 | 1.9 | 11 | 80 |
|  |  | 76.3095 | 23.783 | 20 | 30 | 4.49 | 34.74 | 1.21 | 0.22 | 5.13 | 0.46 | 2.75 | 13 | 2 | Nd | Nd |
| **Stations of E/W transect (Atlantic)** | **Z68** | 76.3333 | 18.7833 | 5 | 5 | 7.18 | 34,00 | 1.94 | Nd | Nd | Nd | Nd | 22 | 2.8 | Nd | Nd |
|  |  | 76.3333 | 18.7833 | 5 | 15 | 7.80 | 34.30 | 1.88 | Nd | Nd | Nd | Nd | 24 | 2.6 | 10 | 85 |
|  |  | 76.3333 | 18.7833 | 5 | 25 | 8.18 | 34.72 | 1.44 | Nd | Nd | Nd | Nd | 21 | 2.8 | Nd | Nd |
|  |  | 76.3333 | 18.7833 | 5 | 35 | 7.29 | 34.87 | 0.32 | Nd | Nd | Nd | Nd | 22 | 2.7 | Nd | Nd |
|  |  | 76.3333 | 18.7833 | 5 | 60 | 6.12 | 34.97 | 0.04 | Nd | Nd | Nd | Nd | 13 | 1.9 | Nd | Nd |
|  | **Z65** | 76.3333 | 14.8833 | 16 | 5 | 6.89 | 34.19 | 1.28 | Nd | Nd | Nd | Nd | 18 | 2.6 | Nd | Nd |
|  |  | 76.3333 | 14.8833 | 16 | 15 | 8.24 | 34.78 | 2.62 | Nd | Nd | Nd | Nd | 21 | 2.7 | Nd | Nd |
|  |  | 76.3333 | 14.8833 | 16 | 25 | 8.17 | 34.84 | 1.62 | Nd | Nd | Nd | Nd | 22 | 2.6 | 11 | 89 |
|  |  | 76.3333 | 14.8833 | 16 | 35 | 7.38 | 34.95 | 0.06 | Nd | Nd | Nd | Nd | 22 | 2.8 | Nd | Nd |
|  |  | 76.3333 | 14.8833 | 16 | 60 | 6.71 | 35,00 | 0.03 | Nd | Nd | Nd | Nd | 19 | 2.6 | Nd | Nd |
|  | **Z61** | 76.3327 | 7.9963 | 16 | 5 | 8.26 | 34.88 | 2.25 | Nd | Nd | Nd | Nd | 22 | 2.8 | 9 | 79 |
|  |  | 76.3327 | 7.9963 | 16 | 10 | 8.24 | 35.04 | 2.3 | 0.05 | 1.35 | 0.26 | 0.9 | Nd | Nd | Nd | Nd |
|  |  | 76.3327 | 7.9963 | 16 | 25 | 8.27 | 34.91 | 2.2 | Nd | Nd | Nd | Nd | 26 | 2.9 | Nd | Nd |
|  |  | 76.3327 | 7.9963 | 16 | 35 | 5.80 | 34.93 | 0.22 | Nd | Nd | Nd | Nd | 21 | 2.5 | Nd | Nd |
|  |  | 76.3327 | 7.9963 | 16 | 60 | 4.53 | 34.99 | 0.08 | Nd | Nd | Nd | Nd | 23 | 2.7 | Nd | Nd |
|  | **Z59** | 76.3317 | 3.9866 | 36 | 5 | 6.11 | 33.49 | 0.61 | Nd | Nd | Nd | Nd | Nd | Nd | Nd | Nd |
|  |  | 76.3317 | 3.9866 | 36 | 22 | 6.06 | 33.64 | 1.92 | Nd | Nd | Nd | Nd | 14 | 2.1 | Nd | Nd |
|  |  | 76.3317 | 3.9866 | 36 | 35 | 3.59 | 34.67 | 2.24 | Nd | Nd | Nd | Nd | 22 | 2.8 | 16 | 50 |
|  |  | 76.3317 | 3.9866 | 36 | 45 | 2.44 | 34.72 | 0.65 | Nd | Nd | Nd | Nd | 22 | 2.6 | Nd | Nd |
|  |  | 76.3317 | 3.9866 | 36 | 60 | 2.83 | 34.84 | 0.17 | Nd | Nd | Nd | Nd | Nd | Nd | Nd | Nd |
