# Supplementary material for: The hydrological context determines the beta-diversity of aerobic anoxygenic phototrophic bacteria in European Arctic seas but does not favor endemism
Source: Front Microbiol. 2015 Jul 3;6:638. doi: 10.3389/fmicb.2015.00638 (PMC4490794; doi:10.3389/fmicb.2015.00638)
Supplement: Supplementary file 2 [file Table2.DOCX]

Table S2: Affiliation of OTUs, their distribution in the libraries and identity (%) of their representative sequences with the next relative sequence and the next relative strain. Phylogroups (E, K, G, and J) correspond to those defined by Yutin et al. [14].

*, Med. Sea, Mediterranean Sea; **NA, not affiliated
